#  Cowpox Virus Transmission from Pet Rats to Humans, Germany 

**DOI:** 10.3201/eid1505.090159

**Published:** 2009-05

**Authors:** Hartmut Campe, Pia Zimmermann, Katharina Glos, Margot Bayer, Hans Bergemann, Caroline Dreweck, Petra Graf, Bianca Kim Weber, Hermann Meyer, Mathias Büttner, Ulrich Busch, Andreas Sing

**Affiliations:** Bavarian Health and Food Safety Authority, Oberschleißheim, Germany (H. Campe, P. Zimmermann, M. Bayer, B.K. Weber, M. Büttner, U. Busch, A. Sing); Haas & Link Tierärztliche Fachklinik für Kleintiere, Germering, Germany (K. Glos); Local Health District Authority, Dachau, Germany (H. Bergemann); Department of Health and Environment, State Capital, Munich, Germany (C. Dreweck, P. Graf); Bundeswehr Institute for Microbiology, Munich (H. Meyer); 1 These authors contributed equally to this article.

**Keywords:** Zoonoses, viruses, cowpox, outbreak, pet rats, orthopoxvirus, humans, Germany, expedited, dispatch

## Abstract

We describe a cluster of cowpox virus (CPXV) infections in humans that occurred near Munich, Germany, around the beginning of 2009. Previously, only sporadic reports of CPXV infections in humans after direct contact with various animals had been published. This outbreak involved pet rats from the same litter.

Cowpox virus (CPXV) belongs to the family *Poxviridae*, genus *Orthopoxvirus* (OPV), and is closely related to other species, such as variola virus, vaccinia virus (VV), and monkeypox virus. Originally, cows were wrongly presumed to be CPXV reservoirs. Wild rodents are now considered to be the true reservoirs; cows, cats, zoo animals, and humans are only incidental hosts (*1*). Human CPXV infections are rare and usually cause localized skin lesions. However, in immunocompromised patients, severe generalized skin infections may occur (*2*). CPXV is transmitted to humans by direct contact with infected animals, mostly cats (*3**–**5*). In 2002, Wolfs et al. described a human CPXV infection transmitted by a wild rat (*6*). We report an epidemiologically linked cluster of 5 cases of human CPXV infection caused by contact with a litter of pet rats (*Rattus norvegicus*).

## The Study

Within 4 days, 5 patients with skin lesions suggestive of an OPV infection were reported to the Infectious Disease Task Force at the Bavarian Health and Food Safety Authority. Infected patients were from 2 unrelated families living in 2 different counties in the greater Munich area, Germany. The families had bought 1 and 2 rats, respectively, from the same pet shop on December 15 and December 17, 2008. Source tracing showed that the pet shop owner had sold a litter of 8 rats to 7 different households in the greater Munich area. The pet shop owner had purchased the litter from a Bavarian rat breeder 7 days before the last rat in the litter was sold. These rats had been kept in cages separate from animals of different species. No symptoms of OPV infection were reported in the rats or any another animal in the pet shop. Moreover, all pet shop workers remained free of signs and symptoms. The pet shop owner denied purchasing any animals from abroad that could have been related to the 2003 US monkeypox outbreak (*7*), although he did acknowledge owning another breeding facility in the Czech Republic. Inspection of the breeding facility in Bavaria found 4 rats with crusts suspicious for OPV infection. Mice, hamsters, rabbits, and degus (*Octodon degus*) were also bred in the facility, but none had clinical signs of OPV. A total of 31 rats from the facility were tested for OPV infection by oral swabs and serology.

**Figure 1:**
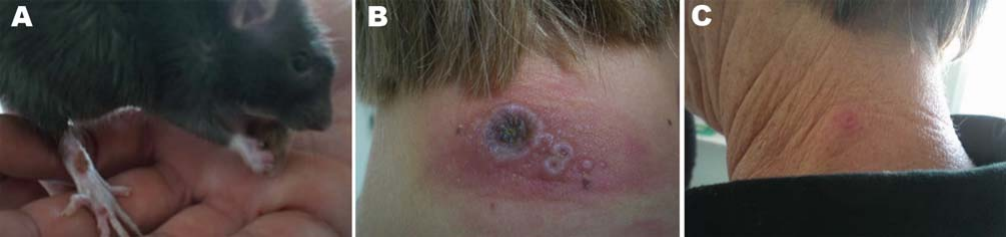
Cowpox lesions on rats and humans during an outbreak in Germany, 2009. A) Rat named Shiva (strain named after this rat) with lesions on the right hind limb; it died 1 day later. B) Neck lesions of a girl without previous vaccinia virus (VV) vaccination. C) Neck lesion of the girl’s grandmother with a history of VV vaccination. Photographs taken by authors 13 days after purchase of the rats. Patient is the grandmother (patient no. 4); rat is rat no. 2.

Members of 6 households were interviewed; 1 customer gave a wrong address. According to their owners, all pet rats were asymptomatic when purchased, but 2 rats (1 in each family with a human OPV infection) died after 9 and 14 days, respectively. One rat had distinct skin lesions on its extremities, mouth, and nose (Figure 1, panel A); the other had only 2 very small lesions on its front leg and nose. Two additional rats from the litter in 2 other households were euthanized due to clinical suspicion of OPV infection; 3 rats were assessed as healthy by their owners.

In households 1 and 2 (Table) with human cases of infection, 2 and 3 persons, respectively, reported only direct skin contact with their pet rats since the first day of purchase. All patients had circumscribed nodules with central necrosis and inflamed edges. Skin lesions were up to 1.5 cm in diameter. Notably, the onset and severity of symptoms were apparently associated with a patient’s VV vaccination status: 2 girls (patients 2 and 5, each 16 years of age with no history of VV vaccination) had multiple lesions on the neck, chest, and abdomen (Figure 1, panel B) accompanied by fever and local lymphadenopathy. Incubation periods for these 2 patients were 3 and 5 days, respectively. In contrast, the incubation period for 2 VV-vaccinated mothers (patients 1 and 3, 42 and 40 years of age, respectively) and for the VV-vaccinated grandmother of 1 of the girls (patient 4, 60 years of age) was >7 days. All showed less severe symptoms (Figure 1, panel C) without fever or lymphadenopathy and only 1 small skin lesion on the neck or chest.

**Table:** Summary of investigations for cowpox virus, by source, household, and individual (human or rat), Munich, Germany, 2009*

Pet origins, households, and cases	Clinical findings	Specimens	Diagnostic methods				
			Antibody titer	EM†	PCR†	Sequencing	Virus isolation
Pet shop							
Household 1							
Human case 1	Lesion	Skin biopsy, serum	640	ND	Positive	Cowpox virus	Positive
Human case 2	Multiple lesions	Serum	2,560	ND	ND	ND	ND
Rat 1	Lesions, fatal outcome	Crusts	ND	ND	Positive	Cowpox virus	ND
Household 2							
Human case 3	Lesion	Crust	ND	ND	Positive	Cowpox virus	Positive
Human case 4	Lesion	NA	ND	ND	ND	ND	ND
Human case 5	Multiple lesions	Crust, serum	1,280	Positive	Positive	Cowpox virus	Positive
Rat 2 and 3	Rat 2: lesion, fatal outcome; rat 3: healthy (no symptoms)	Crusts, serum	Rat 2: 1,280	Rat 2 positive	Rat 2 positive	Rat 2, cowpox virus	Rat 2 positive
Household 3							
Human contact	None	Swabs, blood, serum	Negative	ND	Positive	ND	ND
Rat 4 plus 4 previously owned rats (40–43)	All rats: lesions, euthanized	Crusts	3/3 positive: 160, 1,280, 2,560	ND	5/5 positive	Cowpox virus	ND
Households 4–6							
Human contacts + rats 5–7	All rats and human contacts with no clinical findings; 2 rats euthanized	NA	ND	ND	ND	ND	ND
Breeder							
Rats 9–39	4 rats with lesions (1 dead); all others with no clinical findings	Mouth swabs, serum	4/30 positive (>40)	ND	17/31 positive	Cowpox virus	ND

*All obtained hemagglutinin open reading frames were 924 bp, and the respective sequences were 100% identical to each other (GenBank accession no. FJ654467). Rat 8 from the outbreak litter belonging to household 7 is missing because the rat owner could not be identified. EM, electron microscopy; ND, not done; NA, materials not available. †For *Orthopoxvirus* spp.

In household 3, one person was receiving cyclosporine therapy after a kidney transplantation. She already owned 4 rats before purchasing another rat from the infected litter. After 35 days, skin lesions developed in all of her rats, including the initially asymptomatic index rat. All were euthanized due to clinical suspicion of OPV. Fortunately, the kidney transplant patient without previous VV vaccination did not develop signs or symptoms suggestive of CPXV infection. Nevertheless, we collected a blood sample and swabs from her throat and a recent rat-bite finger wound.

**Figure 2:**
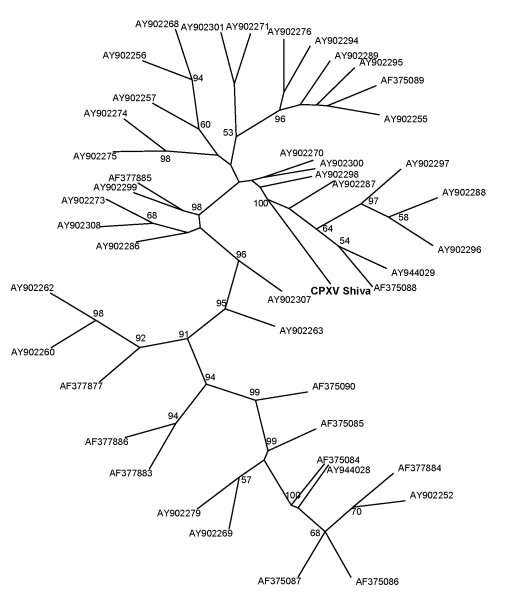
Phylogenetic tree of the isolated cowpox virus (CPXV) Shiva strain (in **boldface**; named after pet rat shown in Figure 1, panel A; GenBank accession no. FJ654467), constructed by the maximum-parsimony method based on the partial sequences method based on the hemagglutinin (HA) gene, unrooted. BLAST search (www.ncbi.nlm.nih.gov/blast/Blast.cgi) confirmed the identification of this strain as a CPXV strain with a unique HA gene sequence. The highest identity of 98.1% was found for strain cowHA72 (accession no. AY902300), a CPXV strain isolated from an elephant in the Netherlands. Bootstrap values >50% are shown. Additional unique CPXV strains shown for comparison, by accession number: AY902307 (cowHA35e), AY902301 (cowHA82), AY902299 (cowHA70), AY902298 (cowHA68), AY902297 (cowHA52), AY902296 (cowHA51), AY902295 (cowHA48), AY902294 (cowHA46), AY902289 (cowHA47), AY902288 (cowHA41), AY902287 (cowHA40), AY902286 (cowHA37), AY902279 (cowHA76), AY902276 (cowHA23), AY902308 (cowHA38), AY902275 (cowHA22), AY902274 (cowHA21), AY902273 (cowHA81), AY902271 (cowHA19), AY902270 (cowHA18), AY902269 (cowHA17), AY902268 (cowHA16), AY902263 (cowHA15), AY902262 (cowHA34), AY902260 (cowHA13), AY902257 (cowHA09), AY902256 (cowHA07), AY902255 (cowHA63), AY902252 (cowHA73), AY944029 (CPV90_ger2), AY944028 (CPV91_ger3), AF377886 (cowpox virus), AF377885 (cpv-922-99), AF377884 (cpv-867-99b), AF377883 (cpv-667-94b), AF377877 (cpv-1218-00), AF375090 (cpx-ep-2), AF375089 (cpx-brt), AF375088 (cpx-90-5), AF375087 (CPX-90-1), AF375086 (cpx-89-5), AF375085 (cpx-89-4), and AF375084 (cpx-89-1).

Various specimens (skin biopsies, crusts, oral swabs, serum, and whole blood) obtained from 5 patients and from rats from 3 households and 31 other rats (9–39) from the local breeding facility in Bavaria were sent to the Bavarian Health and Food Safety Authority (Table). Depending on specimen type, various investigations were performed (Table). Skin biopsy and crust specimens were homogenized and inspected for typical OPV-like particles by using by electron microscopy. Virus isolation for these materials was performed using standard procedures. DNA from all samples was extracted using the QIAamp DNA Mini Kit (QIAGEN, Hilden, Germany) according to the manufacturer’s instructions. For OPV DNA detection, the RealArt Orthopoxvirus LC Kit (QIAGEN) was used. For species identification, the products of a second PCR, spanning the entire open reading frame of the hemagglutinin gene (*8**,**9*), were sequenced. Datasets were edited and aligned using BioEdit (*10*). BLAST search (www.ncbi.nlm.nih.gov/blast/Blast.cgi) was performed to confirm species identification of the isolated strain as well as similarity with published CPXV strains. A phylogenetic tree was constructed using the maximum parsimony method with the Phylogeny Inference Package version 3.68 (http://evolution.genetics.washington.edu/phylip.html) with 100 bootstraps; the tree was drawn with TreeView version 1.6.6 (http://taxonomy.zoology.gla.ac.uk/rod/treeview.html) (Figure 2). OPV-specific serum antibody titers were determined using an immunfluorescence test based on VV-infected cells and either an antihuman or an antirat immunoglobulin G fluorescein-labeled conjugate (Dako, Hamburg, Germany).

## Conclusions

Besides molecularly proven wild rat-to-human CPXV transmission (*6*) an additional CPXV infection probably transmitted from a pet rat was reported (*11*). Recently, 4 human infections acquired from pet rats were reported to the reference laboratory for poxviruses at the Robert Koch Institute (*12*). We describe a CPXV outbreak among 5 patients caused by infected pet rats from the same litter. CPXV infections seem to be increasing (*13*), but because CPXV infections in humans and in most animals (e.g., cats and rats) are not notifiable, this increase remains an assumption. One obvious reason for an increase might be the fading cross-protective immunity to OPV after the cessation of VV vaccination (*14*). In our small cluster, the onset and severity of symptoms seemed to be correlated with VV vaccination status; however, although patients reported similar contact with pet rats, patient age and manner of infection might confound this hypothesis (*15*).

The rising popularity of pet rats might also be a point of concern in a population with decreasing cross-protection to OPV and an increasing number of immunocompromised persons. Our findings emphasize the necessity to monitor OPV infections in humans and all animals (e.g., notification requirement) and to improve public awareness. Our outbreak investigation underlines the importance of close cooperation between human health and veterinary authorities in the management of zoonotic diseases.
